# ElaD, a Deubiquitinating Protease Expressed by *E. coli*

**DOI:** 10.1371/journal.pone.0000381

**Published:** 2007-04-18

**Authors:** André Catic, Shahram Misaghi, Gregory A. Korbel, Hidde L. Ploegh

**Affiliations:** 1 Immunology Program, Harvard Medical School, Boston, Massachusetts, United States of America; 2 Whitehead Institute for Biomedical Research and Department of Biology, Massachusetts Institute of Technology, Cambridge, Massachusetts, United States of America; Institute of Human Virology, United States of America

## Abstract

**Background:**

Ubiquitin and ubiquitin-like proteins (Ubl) are designed to modify polypeptides in eukaryotes. Covalent binding of ubiquitin or Ubls to substrate proteins can be reversed by specific hydrolases. One particular set of cysteine proteases, the CE clan, which targets ubiquitin and Ubls, has homologs in eukaryotes, prokaryotes, and viruses.

**Findings:**

We have cloned and analyzed the *E. coli* protein elaD, which is distantly related to eukaryotic CE clan members of the ULP/SENP protease family that are specific for SUMO and Nedd8. Previously misannotated as a putative sulfatase/phosphatase, elaD is an efficient and specific deubiquitinating enzyme *in vitro*. Interestingly, elaD is present in all intestinal pathogenic *E. coli* strains, but conspicuously absent from extraintestinal pathogenic strains (ExPECs). Further homologs of this protease can be found in Acanthamoeba Polyphaga Mimivirus, and in Alpha-, Beta-and Gammaproteobacteria.

**Conclusion:**

The expression of ULP/SENP-related hydrolases in bacteria therefore extends to plant pathogens and medically relevant strains of *Escherichia coli, Legionella pneumophila, Rickettsiae, Chlamydiae*, and *Salmonellae*, in which the elaD ortholog sseL has recently been identified as a virulence factor with deubiquitinating activity. As a counterpoint, our phylogenetic and functional examination reveals that ancient eukaryotic ULP/SENP proteases also have the potential of ubiquitin-specific hydrolysis, suggesting an early common origin of this peptidase clan.

## Introduction

Ubiquitin, as well as Ubls such as Nedd8 and SUMO, are proteins (almost) exclusively expressed by eukaryotes [1]. Ubiquitination controls many cellular processes, including degradation of proteins by the proteasome and intracellular trafficking. Nedd8 is a ubiquitin-like modifier that regulates the rate or extent of ubiquitination, whereas SUMO1 is involved mostly in regulation of transcription factors and in nuclear import. The attachment of ubiquitin or ubiquitin-like modifiers to substrate proteins is covalent, yet reversible [2]. A large family of eukaryotic cysteine proteases is involved not only in generation of ubiquitin(-like) proteins from their precursors, but also in their removal from modified substrates [3], [4]. Pathogens can tamper with the ubiquitin-proteasome system to cripple the cell's defenses [5]. For instance, ubiquitination and proteasomal degradation of p53, initiated by a Human Papillomavirus protein [6], or stabilization of Iκb-α by *Yersinia* deubiquitinases [7] have been described. The continuing discovery of new deubiquitinating proteases in viruses broadly hints at how important it is for these pathogens to seize control of posttranslational modifications in host cells [8]–[10]. Here, we focus on cysteine proteases of the CE clan [11], as defined by the MEROPS database [12]. CE peptidases are expressed by viruses, bacteria and eukaryotes. In eukaryotes, this clan represents the family of Ubl-specific proteases (ULP/SENP), which remove SUMO or Nedd8 from substrate proteins [13], [14]. Viral homologs of ULP/SENPs can act as deubiquitinases, but they also cleave unrelated proteins, as long as a glycine motif is present at the C-terminus of the substrate [15], [16]. Examples of bacterial deubiquitinases include YopJ and *Chla*DUBs. First, YopJ is a protease that is secreted into host cells by *Y. pestis* [17], and homologs to this peptidase can be found in other bacteria, too [18], [19]. Injection of YopJ eventually suppresses the inflammatory response in affected cells. The precise molecular mechanism of YopJ family peptidases has not yet been solved, as they lack a hallmark tryptophan following the active-site histidine [20]. Yet, *in vitro* and *in vivo* data strongly suggest that these proteins are indeed proteases with specificity for ubiquitin or SUMO [7], [18], [19], although their effectiveness as virulence factors might depend on additional functions such as acetylation [21], [22]. A second example of bacterial CE peptidases can be found in pathogenic *Chlamydiae*. We have shown that pathogenic *Chlamydiae*, but not a non-pathogenic environmental strain, express proteases that specifically recognize ubiquitin and Nedd8. They do so presumably to remove both modifiers from target proteins of the host cell, as *Chlamydiae*–like most other bacteria-possess neither a ubiquitin nor a Nedd8 homolog [23].

Intrigued by the finding of CE peptidases in bacteria and considering their functional similarity to eukaryotic ULP/SENP proteases, we explored whether additional bacterial homologs with deubiquitinating activity might exist.

## Results

**Figure 1:**
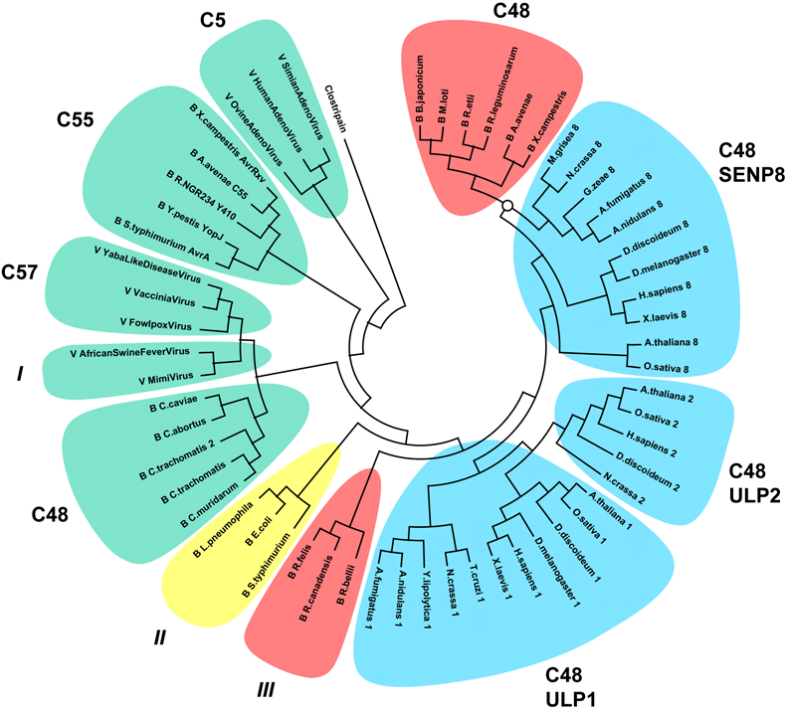
Phylogram representation of CE clan proteases in viruses, bacteria, and eukaryotes. Eukaryotic peptidases (in blue) belong to the C48 subfamily and can be separated into three groups: ULP1 (including the mammalian proteases SENP1, 2, 3, and 5), ULP2 (including SENP6 and 7), and the SENP8 group with proposed specificity for SUMO (ULP1 and ULP2 group) and Nedd8 (SENP8 group), respectively. Bacterial proteins are indicated with a preceding “B”, viral proteins with a “V”. We have further divided microbial protease homologs by color: green for biochemically tested proteases, red indicating the absence of published data on the function of these putative proteases, and yellow for the group representing elaD and its orthologs. The C5 family contains Adenovirus proteases with deubiquitinating activity, C55 comprises the bacterial YopJ homologs, and C57 the Vacciniavirus I7 peptidases. Based on sequence similarity, two bacterial C48 family groups can be distinguished: a group of Proteobacteria (located at one o'clock) which appear to be closely related to fungal SENP8 homologs (common node indicated with a circle, bootstrap support>60%), and *Chlamydiae*, for which we had previously shown the presence of deubiquitinating and deneddylating activity. Three additional groups have not yet been assigned to specific CE clan subfamilies in the MEROPS database, including Mimivirus (“group I”), Gammaproteobacteria (“group II”), and *Rickettsiae* (“group III”). The African Swine Fever Virus protease and the I7 Vacciniavirus protease have not been tested for deubiquitinating or Ubl-specific activity, but they both require a glycine-based motif at the C-terminus of the substrate, as found in ubiquitin or Ubls. The unrelated CD clan peptidase Clostripain is used as outgroup in this phylogram. For clarity, this tree does not contain all orthologs and paralogs of the different groups or families. Sequence information is provided in Table 2.

To search the sequenced genomes of bacteria for new members of the CE protease clan, we employed PHI-BLAST [24] to find proteins with the typical catalytic triad of histidine, aspartate (or glutamate or asparagine), and the active-site cysteine [25]. Candidate proteins were subjected to a second round of analysis, in which we excluded proteins without the hallmark oxyanion-stabilizing group, consisting of at least one glutamine (or asparagine) close to the active-site cysteine. Lastly, the predicted secondary structure of candidate proteases was compared to the solved structure of eukaryotic CE clan homologs [26], [27]. Apart from YopJ and the ULP/SENP homologs in *Chlamydiae*, we found potential CE peptidases in Alpha-, Beta-, and Gammaproteobacteria (Figure 1), but not in bacteria from other branches. We also found a peptidase homolog in the giant Acanthamoeba Polyphaga Mimivirus [28] and in African Swine Fever Virus, the first virus in which a ubiquitin conjugating enzyme was discovered and for which deubiquitinating activity has been suggested [29]–[32]. The bacteria we identified share a close (symbiotic or pathogenic) relationship with eukaryotes, and all viruses with CE proteases possess a dsDNA genome. The sequence variations among these peptidases are too extensive to allow for significant bootstrap values and it is therefore not possible to faithfully infer phylogeny from this dataset. Yet, this is also true for the eukaryotic ULP/SENPs, which fall into three functional classes, specific for either SUMO (ULP1 and ULP2 group) or Nedd8 (SENP8 group). While best reciprocal BLAST hits and consensus phylogram trees tend to correctly predict to which functional class a particular ULP/SENP homolog belongs, bootstrap support is generally not significant [33].

**Figure 2:**
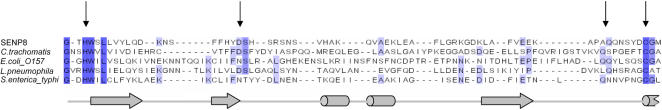
Sequence comparison between SENP8 and its homologs in human pathogenic bacteria. Multiple sequence alignment of the catalytic core region of human SENP8 (NCBI protein sequence identifier GI: 33942066, shown are residues 100–165) with the homologs in *C. trachomatis* (CT868, GI: 76789615, residues 199–284) [23], *E. coli* (elaD, GI: 15832411, residues 228–319), *L. pneumophila* (GI: 52843101, residues 189–265), and *S. typhi* (sseL, GI: 29141091, residues 197–264) [42]. The arrows indicate active-site histidine, aspartate (or asparagine), the catalytic cysteine, and the oxyanion-stabilizing group. Predicted secondary structures are shown at the bottom and have been confirmed with the solved structure of SENP8 [27].

We were especially interested in the protein elaD (belonging to “group II”, see Figure 1), expressed by *E. coli* and with orthologs in *Legionella pneumophila* and in all currently sequenced strains of *Salmonella* (Figure 2). *E. coli* is an abundant commensal in the human gut and also a relevant pathogen. Nonetheless, little is known about genes that define pathogenicity of various *E. coli* strains, apart from those that encode obvious toxins [34]–[36]. We furthermore chose elaD, because it represents one of the more distantly related hits in our bioinformatics screen and we aimed to test the robustness of our prediction by examining this protein's function.

**Figure 3:**
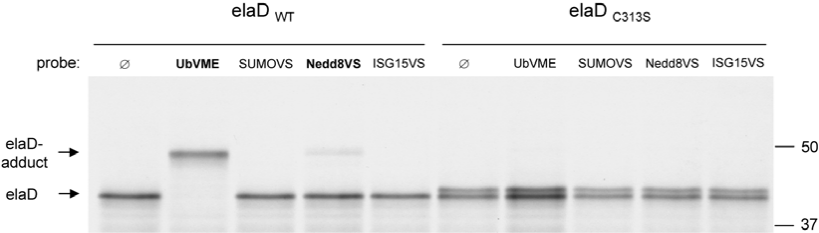
Biochemical assay for substrate specificity of elaD. ^35^S-methionine-labeled *in vitro* translated wildtype elaD forms covalent adducts with suicide inhibitors based on ubiquitin (ubiquitin-vinylmethylester, VME) and Nedd8 (Nedd8-vinylsulfone, VS), but not with probes based on SUMO1 and ISG15. All probes were tested for activity with *bona fide* substrates (not shown) [14]. Mutation of the active-site cysteine at position 313 to serine abolishes adduct formation of elaD to electrophilic probes. Samples were resolved by reducing SDS-PAGE and visualized by fluorography. Indicated at the right is the molecular mass in kDa.

The goal of our first experiment was to confirm protease activity and to determine the substrate specificity of elaD. Because of its relationship to ULP/SENPs, we hypothesized that elaD might recognize Ubls or ubiquitin. We expressed elaD by *in vitro* transcription/translation in rabbit reticulocyte lysate and incubated the metabolically labeled polypeptide with electrophilic probes, in which a Michael acceptor was added to the C-terminus of ubiquitin or the Ubls SUMO1, Nedd8, and ISG15 [14]. As shown in Figure 3, elaD readily forms a covalent adduct with the ubiquitin probe and to a much lesser extent also with the Nedd8 probe, but not detectably with SUMO1 or ISG15. Moreover, when mutating the putative active-site cysteine at position 313 to serine, covalent binding to the electrophile is abolished. This indicates that the cysteine residue in elaD is essential for catalytic activity, as has been observed for YopJ [7], for the *Chlamydia* protease CT868 [23] and for the eukaryotic ULP/SENPs [2]. To date, specific labeling of putative proteases with activity-based probes has shown excellent correlation with enzymatic activity [14], [37], [38].

**Figure 4:**
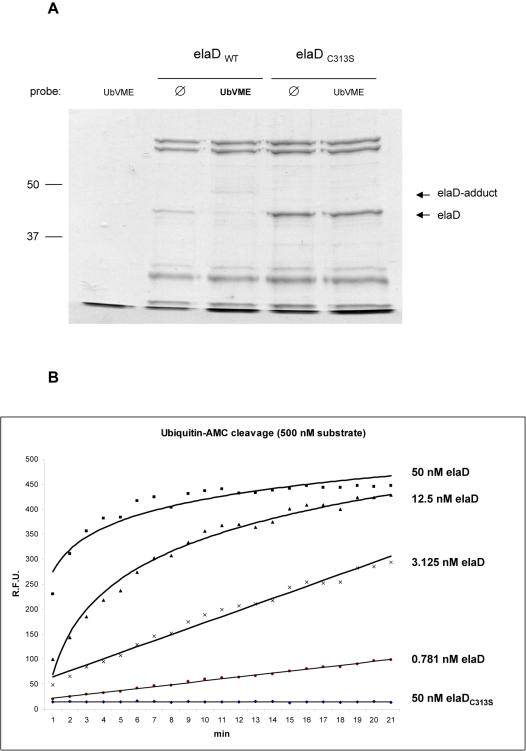
A. Expression of recombinant elaD and elaD_C313S_ in bacteria. Coomassie staining of a 10% SDS-PAGE, showing the relatively higher expression levels of elaD_C313S _, compared to wildtype elaD. The purity of elaD was increased to 30–40% after a second round of size exclusion chromatography (not shown). Similar to *in vitro* translated elaD, the bacterially expressed protein also reacts with ubiquitin-VME. Molecular mass indicated at the left (in kDa). B. Biochemical assay for hydrolytic activity of elaD. His-tagged elaD was expressed in and purified from *E. coli* and incubated with fluorogenic substrates based on ubiquitin, SUMO1 and Nedd8. No hydrolytic activity of elaD was observed on SUMO1-AMC and Nedd8-AMC (data not shown). In contrast, the C-terminal peptide bond of ubiquitin-AMC is efficiently cleaved by elaD, leading to release and dequenching of the fluorophore AMC. Depicted is a representative experiment in which variable concentrations of purified elaD were incubated with 500 nM ubiquitin-AMC for 40 min. The C313S mutant of elaD has no proteolytic activity and wildtype elaD can be inhibited by alkylation of its active-site cysteine with 5 mM N-ethylmaleimide (data not shown). The y-axis shows relative fluorescence units, the x-axis represents the time scale with measurements every 2 minutes.

Next, we assessed enzyme kinetics by measuring hydrolysis of fluorogenic substrates derived from ubiquitin, SUMO1 and Nedd8. For these experiments, we expressed elaD in *E. coli*. The growth rate of the bacteria was unaffected when overexpressing elaD, but we only recovered about 50% of the wildtype protein when compared to the amounts of C313S mutant (Figure 4A). As demonstrated by release of the fluorophore 7-amino-4-methylcoumarin (AMC), elaD cleaves ubiquitin-AMC, but not SUMO1-AMC or Nedd8-AMC, and the C313S mutant of elaD fails to cleave either substrate (Figure 4B and data not shown). The initial rate of hydrolysis with 100 nM ubiquitin-AMC and 50 nM elaD is in the order of 0.3–0.6 per minute, defining elaD as a moderately active deubiquitinase, compared to the rapid Isopeptidase T with a rate of ca. 8 per minute [39] or to the much slower ubiquitin-protease USP14 with a rate of<0.01 per minute (data not shown). It should be noted that we could not assess V_max_, because the enzymatic rate increased linearly with substrate concentration (we tested up to 20 µM ubiquitin-AMC; 50 nM elaD then hydrolyzed ubiquitin-AMC at an initial rate of 5 per minute). Similar observations have been made with the SARS virus deubiquitinase [8]. Overall, our functional analyses confirm the prediction of our bioinformatics screen and define elaD as a deubiquitinating protease.

**Figure 5:**
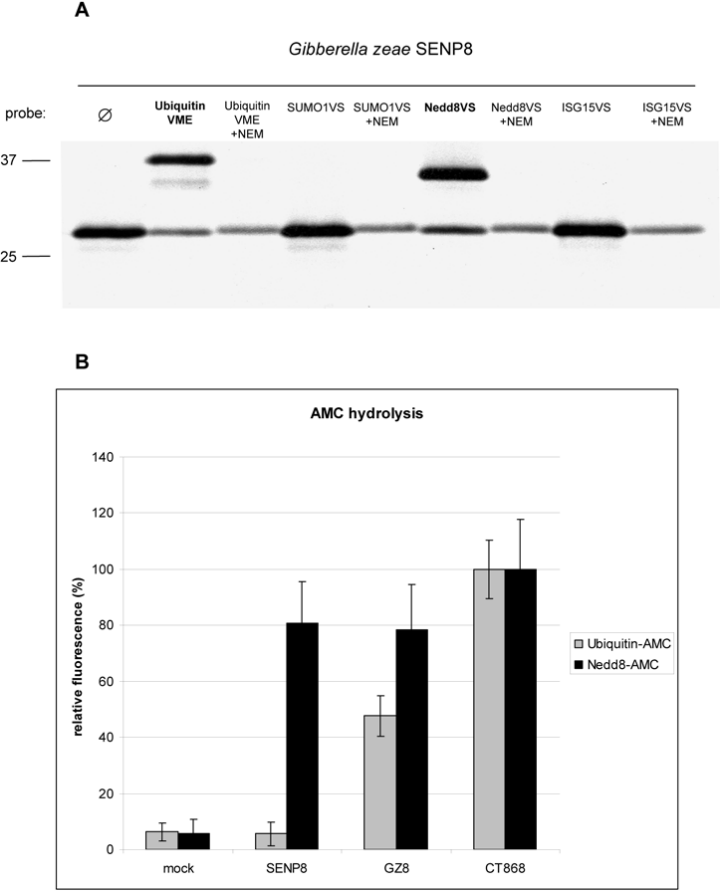
A. Biochemical assay for substrate specificity of GZ8, the SENP8 homolog of the fungus *G. zeae.* Like the chlamydial deubiquitinase/deneddylase CT868 [23], GZ8 reacts with the electrophilic traps ubiquitin-vinylmethylester and Nedd8-vinylsulfone, but not with SUMO1-vinylsulfone or ISG15-vinylsulfone. Shown is SDS-PAGE and fluorography of ^35^S-Methionine-labeled *in vitro* translated GZ8. The cysteine-alkylating agent N-ethylmaleimide (NEM) abrogates activity of the cysteine protease GZ8. Molecular weight markers are indicated at the left (in kDa). B. Biochemical assay for hydrolytic activity of GZ8. Like CT868, but unlike human SENP8, GZ8 exerts hydrolytic activity on ubiquitin-and Nedd8-AMC conjugates. Fluorescence measurement was assayed 16 hours after incubation of human SENP8, GZ8, and chlamydial CT868 (values defined as 100%) with ubiquitin-AMC and Nedd8-AMC. GZ8 and CT868 hydrolyze both substrates, whereas human SENP8 only cleaves Nedd8-AMC. Hydrolysis is sensitive to NEM treatment (not shown). The y-axis depicts relative fluorescence. Shown are the mean values of a representative experiment with triplicate measurements+/−standard deviations.

Why do eukaryotic CE peptidases show specificity distinct from their prokaryotic counterparts? Could this indicate a profound discrepancy, hinting towards a separate origin of these two protease groups? We set out to challenge the presently held notion that ULP/SENP proteases do not exhibit ubiquitin-specific activity [13], while most tested bacterial homologs apparently do. To this end, we chose to biochemically define CE clan members of a more deeply branched class of eukaryotes. Pezizomycotina, a subgroup of fungi that includes *A. fumigatus, A. nidulans, M. grisea, N. crassa*, and *G. zeae* encode putative SENP8 proteases that are related in amino acid sequence to a group of yet uncharacterized bacterial C48 homologs (Figure 1). In our phylogram, the common node between the respective prokaryote C48 group and SENP8 homologs of Pezizomycotina replicates with a bootstrap support of>60%. We cloned, expressed and tested the putative SENP8 protease of *G. zeae*, as a representative of Pezizomycotina. Unlike mammalian SENP8, the *G. zeae* ortholog displays dual activity towards Nedd8 and ubiquitin, similar to the previously defined CE clan protease CT868 of the prokaryote *C. trachomatis* (Figure 5) [23]. This result indicates that ubiquitin-specificity is not restricted to bacterial or viral CE peptidases, but also exists in some ancient eukaryotic members of this protease clan.

## Discussion

We have found previously undescribed CE peptidase homologs in several bacterial and in one viral species (Figure 1). We have furthermore proven that one of the more distantly related homologs–the protein elaD in *E. coli*–can act as a deubiquitinating enzyme *in vitro*. Earlier, we had shown that related *Chlamydia* proteases also have deubiquitinating activity and similar observations had been made regarding the YopJ protease family. This suggests that the yet undefined bacterial CE peptidases could also display ubiquitin-or Ubl-specificity, especially considering their even closer sequence relationship to eukaryotic ULP/SENP proteases. These findings raise two important questions. First, are these proteases really specific for ubiquitin *in vivo*, or are we simply measuring an off-target artifact, with the true substrates merely resembling ubiquitin? Second, why are these proteases-which are present in every eukaryote-so widely distributed in bacteria and viruses as well, and what can we learn about their genetic origin?

One might argue that the bacterial CE clan peptidases have distinct specificity for bacterial substrates. For instance, it has been proposed that the origin of the ubiquitin system predates the split between eukaryotes and prokaryotes [40], [41]. Factors that distantly resemble Ubls and their conjugating and deconjugating enzymes can be found in bacteria. In this manner, the cleavage of ubiquitin by elaD could just be an artificial byproduct of our assays, while the true substrate is of bacterial origin. Similarly, the Adenovirus CE protease has relaxed specificity for a consensus site that is present in ubiquitin, but also in certain viral substrates [15], [16]. However, we have extended our analysis of elaD's specificity to the ubiquitin homologs Nedd8 and ISG15. Both share significant sequence similarity to ubiquitin, and ISG15 is even identical at the critical C-terminal region. We observed no reactivity between elaD and ISG15-vinylsulfone, and the binding of elaD to Nedd8-vinylsulfone was significantly weaker than to the ubiquitin probe. Furthermore, we detected hydrolysis of the C-terminal peptide bond in ubiquitin-AMC, but not in Nedd8-AMC. These features clearly distinguish elaD from the more promiscuous viral CE peptidases. Given the similarity of primary, secondary and tertiary structure among these Ubls, we conclude that hydrolysis of ubiquitin by elaD reflects a highly specific interaction. With the exception of *A. avenae*, no bacterial strain in our dataset encodes a homolog of ubiquitin, making it likely that the substrate of elaD is indeed eukaryotic ubiquitin. Moreover, the ortholog of elaD in *Salmonella*–sseL-has recently been shown to be a virulence factor and to display deubiquitinating activity *in vitro* and *in vivo* [42]. This enzyme is encoded by all currently sequenced *Salmonellae*, but only present as a pseudogene in *Shigella*e [43]. Likewise, elaD is not essential for *E. coli* under laboratory conditions [44]. A comparison of the genomes of all 16 sequenced *E. coli* strains reveals that elaD is present in the commensal *E. coli* strain K12, and in all intestinal pathogenic strains (EAEC, EHEC, EIEC, EPEC, ETEC), but absent from all ExPEC strains (APEC, NMEC, UPEC) (Table 1).

**Table 1:** Expression profile of elaD in different *E. coli* strains.

Group	Strain	elaD
EAEC	101-1	Yes
EHEC	O157:H7 EDL933	Yes
	O157:H7 Sakai	Yes
EIEC	53638	Yes^*^
EPEC	B171	Yes
	E22	Yes
ETEC	E110019	Yes
	E24377A	Yes
commensal	K12/W3110	Yes
	HS	No (pseudogene)
APEC	APEC O1	No
NMEC	O18ac:H7 K1 RS218	No
UPEC	536	No (pseudogene)
	CFT073	No
	F11	No (pseudogene)
	UTI189	No

The gene encoding elaD can be found in all intestinal pathogenic *E. coli* strains, and in the commensal strain K12. The gene is absent in APEC, NMEC, and two uropathogenic strains, and encoded as a pseudogene in three other strains. Interestingly, all pseudogenes in *E. coli* and *Shigellae* begin with the same single-nucleotide deletion, resulting in a frame shift and downstream sequence deterioration. Formally, the strain 53638 also encodes elaD as a pseudogene (indicated with an asterisk), but careful inspection suggests that this is just the result of sequencing slippage in an adenosine-rich region of the elaD gene. APEC, NMEC, and UPEC belong to the group of extraintestinal pathogenic strains (ExPEC).

To answer the second question raised above, the fact that all CE proteases show the same signature motifs at the catalytic domain and that they have substrate specificity for ubiquitin, Ubls, or related products, suggests a common genetic and functional origin of these proteases. The viral and bacterial organisms that express these CE proteases all share an intimate relationship with eukaryotes, either as commensals, symbionts or as pathogens. Additionally, lateral gene transfer has been proposed between eukaryotes and dsDNA viruses [28], as well as between eukaryotes and bacteria such as *Chlamydiae, Rickettsiae*, and *L. pneumophila* [45], [46]. The notion of a dynamic horizontal gene transfer involving deubiquitinases is further underscored by the distribution of ubiquitin-specific proteases in *Chlamydiae*: all pathogenic strains express CE peptidases, except for *C. pneumoniae*, which instead encodes a homolog of the eukaryotic otubain-type deubiquitinating enzymes [47]. Also, one group of bacterial C48 peptidases in particular clusters close to the SENP8 homologs of Pezizomycotina (Figure 1). The sequence relationship and the shared habitat of these organisms-plant symbionts and phytopathogens vs. environmental fungi-raises the possibility of gene transfer between them [48]. From a functional perspective, we could show that a homolog of SENP8 in Pezizomycotina does exert ubiquitin-specific hydrolase activity, like previously characterized bacterial CE proteases (Figure 5). The dual specificity of SENP8 from the fungus *G. zeae* towards ubiquitin and Nedd8 is not trivial. Although Nedd8 is arguably the closest relative to ubiquitin, there are sequence differences that require distinct conjugating machineries and deconjugating proteases [27], [49]. In particular position 72–an arginine in ubiquitin, and an alanine in Nedd8–can act as a compatibility switch and a single replacement at this side chain can cause ubiquitin to mimic Nedd8 and vice versa [50], [51]. In this respect, the recognition of both ubiquitin and Nedd8 by the SENP8 homolog of *G. zeae* is in contrast to what has been observed in mammalian SENP8 [27].

One possible explanation for these observations is that CE proteases originally derived from a deubiquitinase, a protease specific for this most conserved eukaryotic protein. As CE peptidases in eukaryotes structurally diversified to accommodate the evolving family of Ubls, protease counterparts in bacteria, viruses, and some deeply branched eukaryotes retained their specificity for the “ur-substrate” ubiquitin.

Together with the published literature, our data supports the notion that the clan of CE proteases was acquired by bacteria and viruses via horizontal gene transfer from eukaryotes. Why this family of enzymes forms such a particularly attractive substrate for genetic exchange is an intriguing question. The distribution of CE proteases in symbiotic and pathogenic prokaryotes and viruses is suggestive of a general role in host-microbe interactions, as exemplified by the *Salmonella* protease sseL [42].

## Materials and Methods

### Phylogenetic Analysis

Protein and DNA sequence data were obtained from the National Center for Biotechnology Information (www.ncbi.nlm.nih.gov), the Institute for Genomic Research (www.tigr.org), and the University of Wisconsin *E. coli* Genome Project (www.genome.wisc.edu). Protein sequence identifiers are listed in Table 2. Sequences containing and surrounding the catalytic core of the proteases were aligned with the ClustalX algorithm (default parameters) (bips.u-strasbg.fr/fr/Documentation/ClustalX) [52], manually edited with Genedoc (www.psc.edu/biomed/genedoc/) (by K.B. Nicholas&HB Nicholas Jr), using the active-site amino acids as anchor, and visualized with JalView (http://www.jalview.org) [53]. Secondary structures were predicted with JPred (www.compbio.dundee.ac.uk/∼www-jpred/) [54]. Phylograms were constructed with the MEGA software package [55], using the Neighbor-Joining Method with Poisson correction (all substitutions, homogeneous pattern, γ-distribution 2.0) and pairwise deletion of gaps. Figure 1 shows a consensus tree based on 100 bootstrap replications.

**Table 2:** NCBI protein sequence identifier numbers of microbial CE peptidase homologs.

Group	Species	NCBI identifier	Subdivision
*I*	Acanthamoeba Mimivirus	55819230	
	African Swine Fever Virus	9628218	
*Chlamydiae*	*C. abortus*	62185296	
	*C. caviae*	29840476	
	*C. muridarum*	15834878	
	*C. trachomatis*	76789615	
	*C. trachomatis* (paralog)	76789616	
*II*	*E. coli*	2498328	Gamma
	*L. pneumophila*	52843101	Gamma
	*S. typhimurium*	16765614	Gamma
*III*	*R. bellii*	91205013	Alpha
	*R. canadensis*	102192100	Alpha
	*R. felis*	67458601	Alpha
*C48 ( putative)*	*A. avenae*	111620428	Beta
	*B. japonicum*	27383355	Alpha
	*M. loti*	20803886	Alpha
	*R. etli*	86359735	Alpha
	*R. leguminosarum*	116255006	Alpha
	*X. campestris*	78045993	Gamma

The putative proteases are separated by groups (as shown in Figure 1), by species, and by subdivision of Proteobacteria. This list is not complete in terms of orthologs/paralogs or bacterial species.

### Cloning, expression, and biochemical analysis of elaD and *G. zeae* SENP8

The full-length elaD gene (NCBI protein sequence identifier GI: 16130204) was amplified by PCR from the K12 strain BL21 (Novagen) and cloned into pET28a (Novagen). PCR was performed with the Platinum Supermix (Invitrogen), following the manufacturer's instructions (T_a_ 55°C, 32 cycles) and with these primers for NdeI/XhoI insertion into pET28a: Forward: 5′ GGGAATTCCATATGATGATGGTTACAGTTGTC AGCAATT 3′; Reverse: 5′ CCGCTCGAGTTAACTCACTCTTTTGCCGGATGC 3′. The elaD_C313S_ mutant was generated with the Phusion Site-Directed Mutagenesis kit (New England Biolabs), according to the manufacturer's suggested protocol with the following 5′ phosphorylated and PAGE-purified primers: Forward: 5′ AGCGGTGCAT TTGTGTGCATGGCAGCC 3′; Reverse: 5′ ACTTTGGCTTAAGTATTGCTGAAG 3′. The cDNA library of nitrogen starved *Gibberella zeae* was obtained from the Fungal Genetics Stock Center (University of Missouri) [56]. The catalytic core region of the *G. zeae* SENP8 homolog (protein sequence identifier GI: 46121295), spanning from residues 1-228, was cloned via PCR and EcoRI/SalI insertion into pET28a, using Platinum Supermix (T_a_ 65°C, 36 cycles) and these primers: Fwd.: 5′ GGCCGGGAA TTCATGCCGTTTCGTCAGAGGATGG 3′; Rev.: 5′ GGCCGGGTCGACTCAGCG TGTTTGGATCACCTTG 3′. The final cloning products were fully sequence confirmed with primers for the T7 promoter and terminator site of pET28a (Novagen primers 69348-3 and 69337-3). All three proteins were expressed in *E. coli* BL21(DE3) (Novagen) after induction with 1 mM IPTG for 3–4 hours at 37°C, and isolated with Nickel NTA-Agarose under native conditions (see “The QIAexpressionist” handbook; http://www1.qiagen.com/HB/QIAexpressionist). The initial purity was 5–10% (elaD and elaD_C313S_) and 70% (*G. zeae* SENP8), respectively and improved after size exclusion chromatography (Superdex75-prep, Amersham) to a final purity of 30–40% (elaD and elaD_C313S_) and>90% (*G. zeae* SENP8), as assessed by Coomassie staining. Importantly, the quality of the final preparations was identical for elaD and elaD_C313S_. For the specificity screen, the proteases were expressed by *in vitro* transcription/translation in reticulocyte lysate (TNT T7 Quick coupled IVT kit, Promega) in the presence of ^35^S-methionine (PerkinElmer NEG709A), and then diluted 3–4×with a buffer containing Tris (50 mM, pH 7.5), NaCl (150 mM) and DTT (2 mM or 5 mM N-ethylmaleimide for the negative control), before 0.1 µg of the electrophilic probes were added for 20–40 min at room temperature. The AMC hydrolysis screen was conducted in the same dilution buffer as described above, with an additional 1 mg/ml bovine serum albumin. 100 nM to 20 µM of ubiquitin-AMC, SUMO1-AMC, and Nedd8-AMC (Boston Biochem) were incubated with various concentrations of bacterially expressed elaD or elaD_C313S_ at 27°C for the indicated time points. The *G. zeae* SENP8 experiments were conducted as endpoint measurements (100 nM AMC substrates, 60 nM protease, and 16 hours reaction time). Fluorescence was analyzed with a Spectramax M2 multi-detection microplate reader (Molecular Devices).
